# Serum concentrations of Thymidine kinase 1 measured using a novel antibody-based assay in patients with Hodgkin Lymphoma

**DOI:** 10.48101/ujms.v126.6119

**Published:** 2021-08-20

**Authors:** Johan Mattsson Ulfstedt, Per Venge, Sofia Holmgren, Gunilla Enblad, Staffan Eriksson, Daniel Molin

**Affiliations:** a Experimental and Clinical Oncology, Department of Immunology, Genetics and Pathology; Uppsala University, Uppsala, Sweden; b Department of Medical Sciences, Uppsala University, Uppsala, Sweden; c Diagnostics Development, Uppsala, Sweden; d Department of Anatomy, Physiology & Biochemistry, Swedish University of Agricultural Sciences, Uppsala, Sweden; e AroCell AB, Uppsala, Sweden

**Keywords:** Hodgkin lymphoma, TK1, Thymidine kinase, prognostic markers, chemotherapy

## Abstract

**Background:**

Thymidine kinase 1 (TK1) is an intracellular protein associated with DNA synthesis, expressed during the G1 phase and remained elevated through the M phase, with a potential as a biomarker for cell proliferation. In this study, we explore the possible use of TK1 in Hodgkin lymphoma (HL).

**Methods:**

Serum concentrations of TK1 (S-TK1) were measured in 46 newly diagnosed HL patients using prospectively collected biobanked serum samples. The samples were analyzed using a novel antibody-based TK1 immunosorbent assay (ELISA).

**Results:**

The concentrations of S-TK1 were elevated in HL patients compared with healthy controls (median 0.32 μg/L vs. 0.24 μg/L, *P* = 0.003). A further increase in S-TK1 was observed during the treatment. The S-TK1 concentrations were higher in patients with advanced stage disease, low B-Hb, elevated P-LD and in those with B-symptoms. A high ESR correlated with low S-TK1.

**Conclusions:**

The study results suggest that S-TK1, measured using a novel antibody-based assay, has the potential to be a biomarker in HL. However, while S-TK1 levels are elevated at baseline compared with healthy controls, a limited number of patients and comparatively short follow-up time render reliable conclusions difficult.

## Introduction

Thymidine kinase (TK) is an intracellular protein associated with DNA synthesis with a potential as a biomarker for cell proliferation. Its use has been explored in several tumor diseases (1). TK exists in two forms: TK1 is present in cytoplasm in a cell cycle dependent manner, while TK2 is located in the mitochondria in all cells. TK1 catalyzes the ATP-dependent phosphorylation of thymidine to thymidine 5ʹ-monophosphate for use in DNA synthesis (2). The expression of thymidine kinase-1 (TK1) rises during the late G1 phase, under the control of the E2F transcription factor, and remains elevated during the S, G2, and M phases. TK1 is subsequently degraded after completion of a controlled cell cycle. TK1 concentrations in serum are thus indirect indicators of disruption of dividing cells through uncontrolled processes such as necrosis.

TK1 is traditionally measured by its enzymatic activity and used clinically in non-Hodgkin lymphoma (NHL) but has been documented in few studies on Hodgkin lymphoma (HL) (3). However, the TK1 molecule is present in serum in different complexes, not all are enzymatically active. To address this issue, AroCell has developed the novel TK1 210 enzyme-linked immunosorbent assay (ELISA) to measure the total content of TK1.

In HL tumors, the malignant Hodgkin and Reed–Sternberg (HRS) cells are limited in number, often making histological diagnosis difficult for pathologists, while the bulk of the tumor consists of normal reactive immune cells. The cell turnover in HL would thus be primarily attributed to apoptosis in healthy cells, with a controlled turnover of cell cycle-dependent proteins, including TK1. Our hypothesis is that TK1 is a more sensitive tumor cell turnover marker compared with lactate dehydrogenase (LD), commonly used as a biomarker in lymphoma. Thus, in this study, we investigated the feasibility of using the cell cycle-dependent TK1 as a possible biomarker and early treatment predictor in a cohort of HL patients.

## Materials and methods

Serum TK1 (S-TK1) was measured in HL patients before and during treatment using the AroCell TK 210 ELISA, according to the manufacturer’s instructions (AroCell AB, Uppsala, Sweden). The technical specifications of the TK 210 ELISA assay, including comparison with the existing Abcam TK1 ELISA assay, have previously been published (4). The TK 210 assays show higher sensitivity and specificity for hematological malignancies, as well as superior discrimination for solid tumors.

Fifty-eight patients with primary or recurrent HL were recruited as part of the Uppsala Umeå Comprehensive Cancer Consortium (U-CAN) project (5) between September 2010 and November 2016, and their characteristics are shown in Table 1. The HL cohort includes patients treated at Uppsala University Hospital and those treated at regional hospitals referred to by Uppsala University Hospital. For logistical reasons and changes in routine sampling points over time, not all patients have a consistent biobank coverage in our cohort. Samples were released from the U-CAN biobank for the TK1 analysis under ethical approval (dnr. 2013/059). For comparison, 269 healthy non-infected controls, recruited as part of a health survey, were analyzed. The within-assay and between-assay imprecisions were found to be between 5% and 8% CV, respectively (6). A total of 135 samples were analyzed. Baseline samples at diagnosis were available for 39 patients and seven at the time of relapse. Sequential samples at baseline and after two cycles of chemotherapy were available for 11 patients in the biobanked material.

**Table 1:** Patient characteristics.

Variable	HL *n* = 58
	Median (range) or *n* (%)
**Age**	42.5 (18–88)
**Sex**	
Female	21 (36)
Male	37 (64)
**Histology**	
Nodular sclerosis	37 (64)
Mixed cellularity	6 (10)
Lymphocyte predominant	2 (3)
Lymphocyte depleted	0 (0)
cHL -NOS*	13 (22)
**Stage**	
I	7 (12)
II	22 (38)
III	16 (28)
IV	13 (18)
**B-symptoms**	
no	36 (62)
yes	22 (38)
**Treatment**	
Adriamycin, Bleomycin, Vinblastine, Dacarbazine	44 (76)
Bleomycin, Etoposide, Adriamycin, Cyclophosphamide, Oncovin, Procarbazine, Prednisone	3 (5)
Cyclophosphamide, Hydroxydaunorubicin, Oncovin, Prednisone variant	7 (12)
Other**	4 (7)
**Radiotherapy**	24 (41)
**Deaths**	
Total	10 (17)
Treatment related	2 (3)
Disease related	4 (7)
Other	4 (7)
**Hemoglobin** (g/L)	130.5 (93–172)
**Leukocyte count** (× 10^9^ /L)	8.85 (4.3–23.9)
**Lymphocyte count** (× 10^9^ /L)	1.5 (0.5–2.8)
**Lymphocyte count** (%)	15.6 (3.9–35.3)
**ESR** (mm/h)	23.5 (2–110)
**C-reactive protein** (mg/L)	27 (0.7–215)
**Albumin** (g/L)	35 (22–46)
**LD** (μkat/L)	3.1 (2.1–9.7)

ESR, erythrocyte sedimentation rate; LD, lactate dehydrogenase.

* Classical HL not otherwise specified histology (cHL-NOS).

** ICE, Bendamustin, Rituximab, CEOP.

Non-parametric statistics were applied unless otherwise indicated. A *P*-value of <0.05 was considered statistically significant. Multiple regression analysis was applied, with S-TK1 as the dependent variable and other numerical serum or plasma variables as independent variables. Statistical analysis was performed using the Medcalc Statistical Software version 19 (MedCalc Software bvba, Ostend, Belgium; https://www.medcalc.org; 2019).

## Results

**Figure 1:**
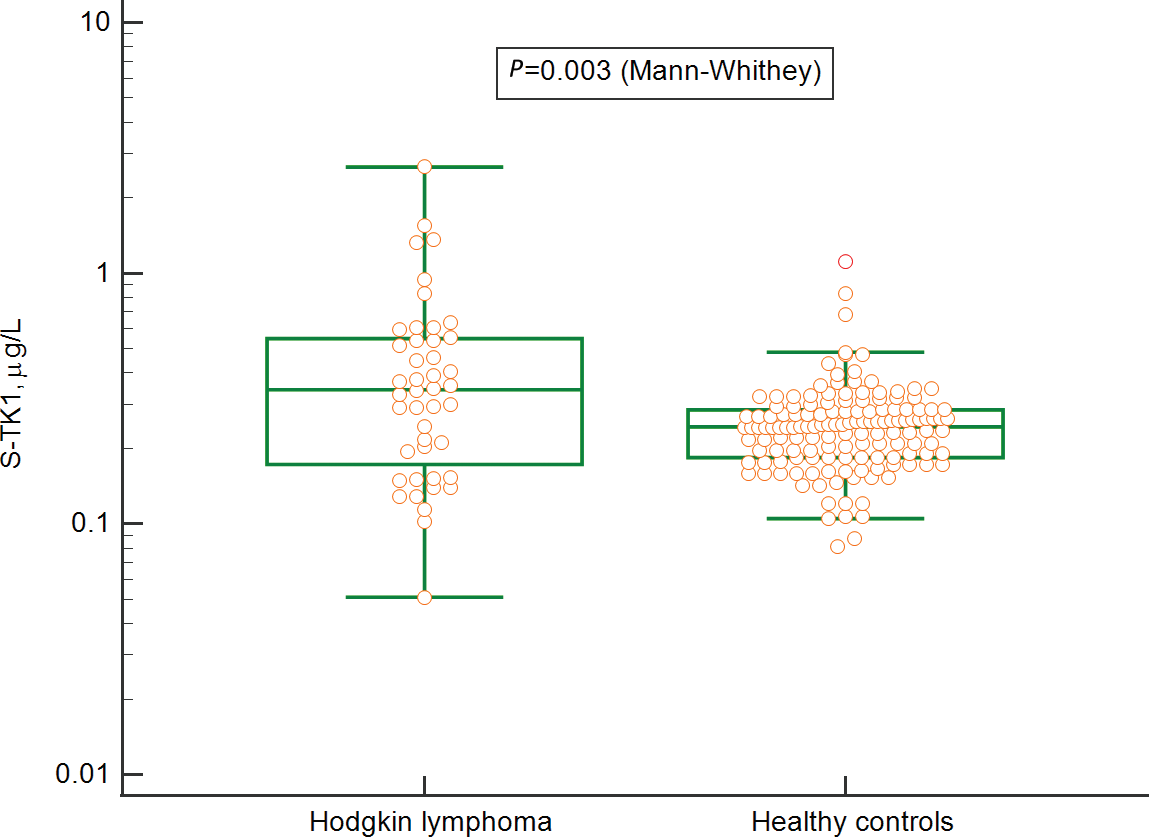
Concentrations of Thymidine kinase 1 in serum of patients with Hodgkin lymphoma before treatment and healthy controls, visualized using a boxplot showing median value, interquartile range (IQR), and whiskers for Q1 – 1.5 • IQR and Q3 + 1.5 • IQR respectively.

The S-TK1 concentrations were elevated in newly diagnosed HL patients when compared with healthy controls (median 0.32 μg/L vs. 0.24 μg/L, *P* = 0.003), with a proposed cut-off of 0.45 g/L based on the upper 97.5 percentile limit of the healthy population (Figure 1).

**Figure 2:**
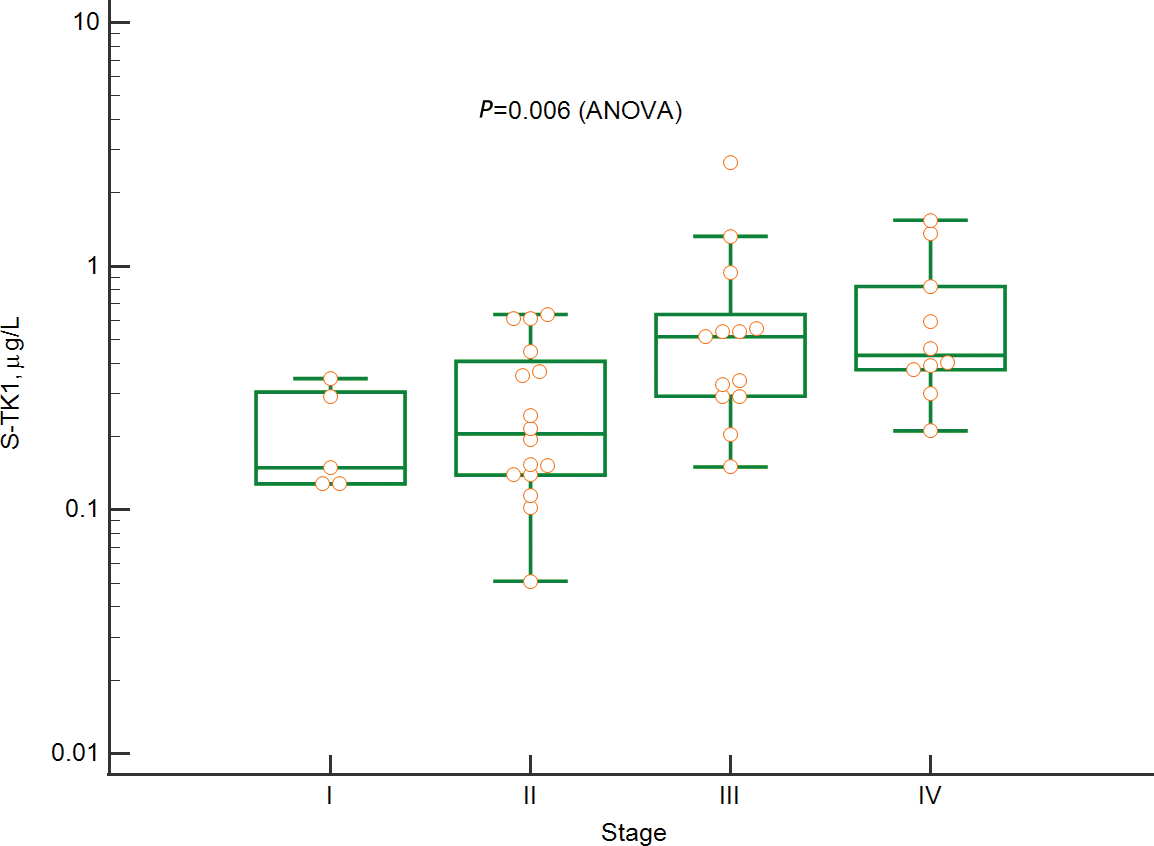
Relation between Thymidine kinase 1 concentrations in serum and disease stage.

High S-TK1 concentrations correlated with stage and were highest in those with stage IV disease (*P* = 0.006, ANOVA) (Figure 2). In addition, high S-TK1 correlated with the presence of B-symptoms (*P* = 0.02) and high international prognostic score (IPS) (*P* = 0.02).

**Figure 3:**
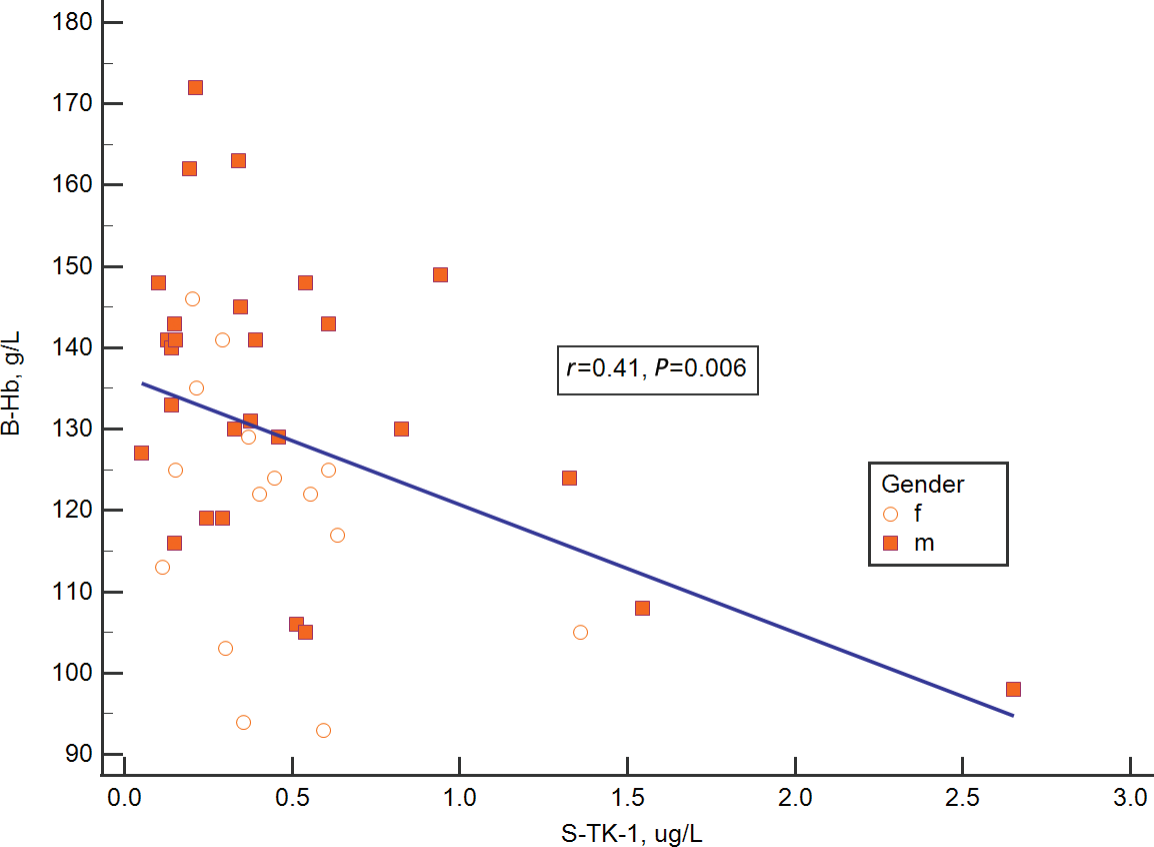
Relation between Thymidine kinase 1 concentration in serum and hemoglobin.

**Figure 4:**
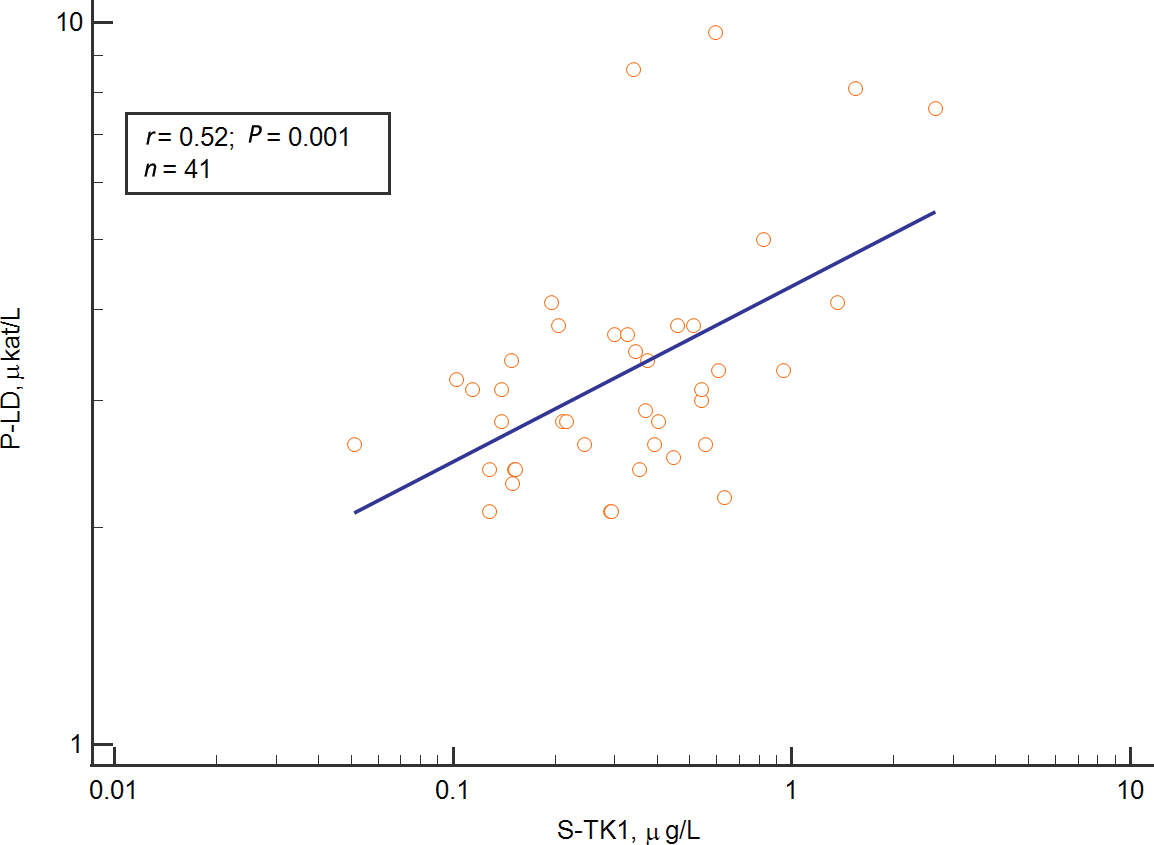
Relation between Thymidine kinase 1 concentrations in serum and Lactate Dehydrogenase.

In a multivariate analysis, high S-TK1 concentrations pre-treatment correlated with low blood hemoglobin (B-Hb) (Figure 3), high plasma LD (P-LD) (Figure 4), and low erythrocyte sedimentation rate (ESR), but not with other variables included in the model, that is, total white blood cells, plasma albumin and plasma C-reactive protein.

**Figure 5:**
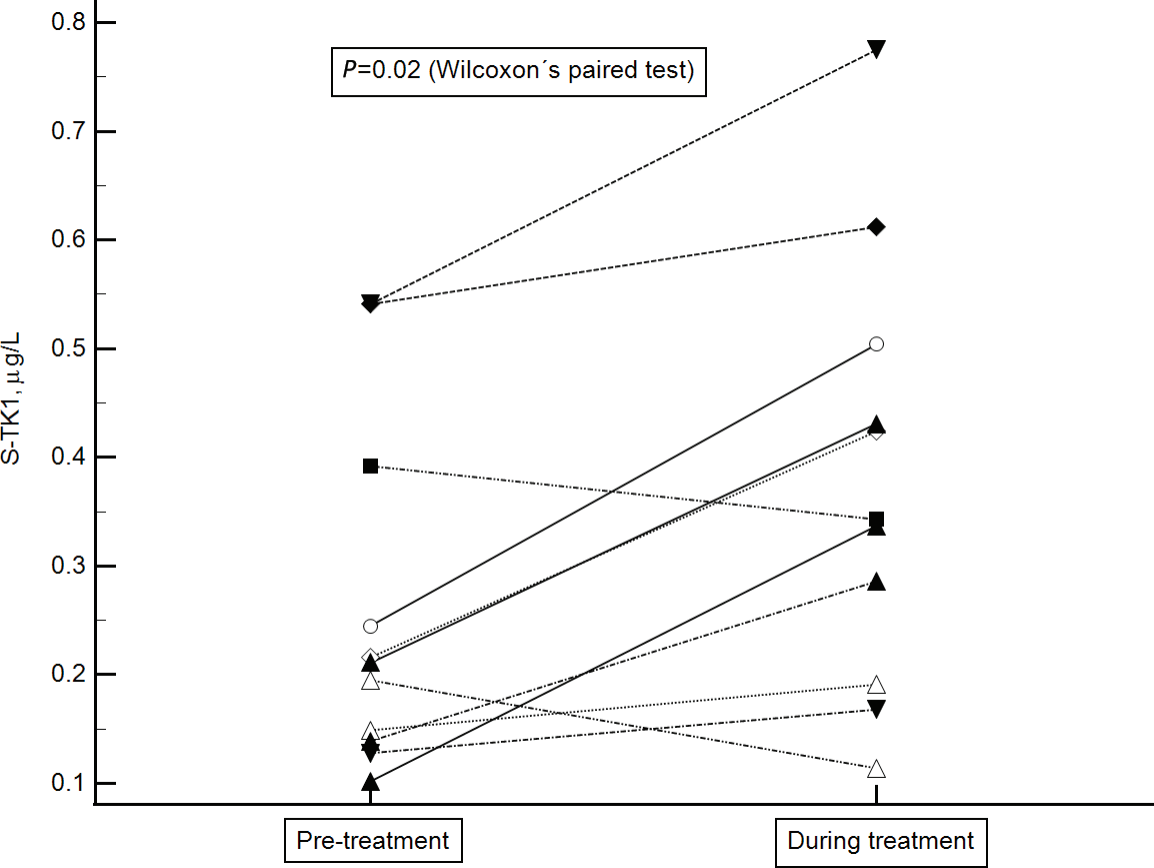
Linked Thymidine kinase 1 concentration changes in serum pre-treatment and during treatment.

During treatment, after two cycles of chemotherapy, the S-TK1 concentrations increased. The increment was observed in 9 of 11 patients for whom data were available (*P* = 0.02, Wilcoxon’s paired test; Figure 5). One patient with increment (one of nine) and one without increment (one of two) showed an inadequate response on interim 2-deoxy-2-[fluorine-18]fluoro-D-glucose positron emission tomography–computed tomography (^18^F-FDG-PET/CT) defined as Deauville score ≥ 4 (7), However, all patients remained relapse free at the time of analysis.

Correlation with overall or progression-free survival could not be determined due to the excellent prognosis of HL and limited follow-up time.

## Discussion

To our knowledge, this research study presents the only analysis of TK1 protein concentrations in the serum of HL patients before and during treatment using a modern antibody-based ELISA assay. In our material of newly diagnosed HL patients, we have found elevated concentrations of S-TK1 compared with healthy controls, with a further increase during treatment. The S-TK1 concentrations were higher in patients with advanced stage, low B-Hb, elevated P-LD and B-symptoms. However, high S-TK1 correlated with low ESR. The correlations to stage, P-LD and B-symptoms as defined by the Ann Arbor staging criteria (8) (an unexplained weight loss of more than 10% of body weight in less than 6 months, unexplained fever with temperatures above 38°C and night sweats) are in accordance with previously published results, where TK was measured by its enzymatic activity (3).

The value of P-LD in NHL was early recognized (9) and is well established (10). P-LD has been evaluated for HL (11), but did not pass the test of time, being replaced by IPS (12). In HL tumors, the malignant HRS cells are very few, while the bulk of the tumor consists of reactive normal immune cells. It is thus likely that the increased concentrations of S-TK1 found in our material reflect the increased cell proliferation in a non-HL specific manner. B-symptoms as an indirect marker for inflammatory activity is an independent prognostic factor for all stages. Inflammatory activity as measured by ESR in HL patients is only a prognostic marker for early stages (I–IIA). In advanced stages (IIB–IVB), a low B-Hb level indicates a chronic inflammatory response associated with poorer prognosis. Thus, S-TK more accurately reflects the cell turnover by the supporting normal immune cells in patients with HL, which is in line with the positive correlation with stage. The contradictory correlations with low B-Hb and low ESR found in this study could indicate that S-TK1 is an independent variable from the inflammatory reactions as measured by ESR in HL. This observation warrants further exploration of the role of S-TK1 in the activation of the micro-environment by the HRS cells.

The results of this study reveal that the elevation of S-TK1 concentrations in HL is modest compared with healthy controls. Values are considerably higher in patients with leukemias, myelomas, myelodysplastic syndromes (4) and diffuse large B-cell lymphoma (DLBCL) (data not yet published). The dynamics during treatment are also more pronounced in DLBCL, where the majority of tumor cells would display an unregulated TK1 expression. In comparison, the cell turnover in HL tissues largely occurs through apoptotic pathways in normal immune cells, producing lower baseline measures. In HL, the expression of TK1 is likely tightly controlled in the majority of cells, thus TK1 release secondary to chemotherapy induced necrosis would be relatively low due to the presence of few HRS over all.

While current stratification of patients with HL follows the IPS (12), it operates on a population basis. As such it does not adequately reflect the individual patient’s prognosis, and thus, highlights the need for new biomarkers. S-TK1, which correlated with low B-Hb and stage IV in the IPS, is such a potential biomarker. However, this feasibility study was not powered to show a correlation to survival.

Interim ^18^F-FDG-PET/CT has been found to be superior in predicting outcomes compared with IPS (13) and measures response to treatment. While ^18^F-FDG-PET/CT in most patients shows a metabolic response after two cycles of chemotherapy, S-TK1 in our material increased during therapy. Given these dynamics of S-TK1, early assessment might be useful, ideally during the first cycle of chemotherapy.

The mechanism for S-TK1 increase during treatment has been established in cell culture experiments measuring TK1 release in cells treated with doxorubicin (14). Chemotherapy agents, such as doxorubicin and dacarbazine, used in HL are known to halt the cell cycle in the G2/M phases, where TK1 is actively expressed. TK1 has also been shown to peak on day 1 after starting treatment in NHL patients (15). The increase of TK1 during therapy in our material is consistent with these findings.

As previous studies have shown that the TK1 values vary over time in relation to the administration of chemotherapy, further studies with repeated analyses are needed to ascertain the best time to analyze TK1 in relation to the administration of chemotherapy. U-CAN samples are collected either midcycle or prior to cycle 3. TK1 concentrations are thus collected at their lowest point in relation to chemotherapy, which strengthens the argument for TK1 as a possible biomarker.

In conclusion, our analysis suggests that relative change in TK1, before and after initiation of chemotherapy could be a potential biomarker in HL; however, further studies are needed to determine the role of TK1 in relation to chemotherapy administration.
